# Single cell RNA-seq identifies a FOS/JUN-related monocyte signature associated with clinical response of heart failure patients with mesenchymal stem cell therapy

**DOI:** 10.18632/aging.205670

**Published:** 2024-03-20

**Authors:** Hui Yuan, Pengfei Zhang, Yuanfeng Xin, Zhongmin Liu, Bingren Gao

**Affiliations:** 1 Department of Cardiac Surgery, Lanzhou University Second Hospital, Lanzhou 730030, Gansu, China; 2 Shanghai East Hospital, School of Medicine, Tongji University, Shanghai 200092, China; 3 Cardiopulmonary Vascular Center, Haikang Hospital, Xingguang Island, West Coast New Area, Qingdao 266400, Shandong, China

**Keywords:** heart failure, MSCs, FOS, JUN, machine learning

## Abstract

Heart failure (HF) is a serious global health issue that demands innovative treatment approaches. In this study, we collected samples from 4 HF patients before and after MSC therapy and performed scRNA-seq. After the MSC therapy, the proportion of CD14^+^ monocytes decreased significantly in both the treatment response and non-response groups, with a more pronounced decrease in the treatment response group. The therapy-response and non-response group were clearly separated in the UMAP plot, while the CD14^+^ monocytes in the therapy-response group before and after MSC therapy were very similar, but there were significant differences in the non-response group. By further performing NMF analysis, we identified 11 subsets of CD14^+^ monocytes. More importantly, we identified a therapy-related CD14^+^ monocyte subpopulation. The predictive model based on CD14^+^ monocytes constructed by machine learning algorithms showed good performance. Moreover, genes such as FOS were highly enriched in the therapy-related CD14^+^ monocytes. The SCENIC analysis revealed potential regulatory factors for this treatment-responsive CD14^+^ monocytes, and FOS/JUN were identified as potential core indicators/regulators. Finally, HF patients were divided into three groups by NMF analysis, and the therapy-responsive CD14^+^ monocyte characteristics were differentially activated among the three groups. Together, this study identifies treatment-responsive CD14^+^ monocytes as a crucial biomarker for assessing the suitability of MSC therapy and determining which HF patients could benefit from it. This provides new clues for further investigating the therapeutic mechanisms of MSC therapy, offering beneficial insights for personalized treatment and improving prognosis in HF patients.

## INTRODUCTION

Heart Failure (HF) is a serious heart disease, and its treatment has always been a major challenge in the field of medicine [1]. Conventional treatment options encompass prescription drugs, revascularization procedures, implantation of ventricular assistance devices, and heart transplantation, though these methods are subject to certain limitations [2]. At present, various types of stem cells can be used for transplantation to treat various diseases, including induced pluripotent stem cells (iPSC) and their differentiated products [3], mesenchymal stem cells (MSC) [4], embryonic stem cells (ESC), and their differentiated products [5]. MSCs were the earliest stem cell type implemented in clinical research due to their relative abundance as they are obtainable from various human tissues, including bone marrow, umbilical cords, fat and so on [6–8].

In recent years, mesenchymal stem cells (MSCs) have become a popular type of cells for clinical translation due to their convenient sourcing, abundant intracellular content in source material, low immunogenicity, ethically uncontroversial acquisition, and strong proliferation capacity [9]. MSCs exhibit extremely low immunogenicity in allogeneic transplantation and have gradually been employed for treating various diseases, particularly in the realm of cardiovascular disorders, notably for addressing congestive heart failure [10–12]. Traditional research shows that the mechanisms underlying stem cell therapy for HF include direct differentiation into cardiomyocytes to replace damaged myocardium, or differentiation into vascular endothelial cells to improve microcirculation, as well as promoting endogenous repair of myocardial cells through mechanical stimulation [13]. Nevertheless, investigators have found that stem cells, particularly MSCs, exert a more substantial paracrine function. For instance, hUC-MSCs exert diverse functions by secreting extracellular vesicles in damaged regions such as myocardium, neurons, skin, and liver, including anti-apoptotic effects, promotion of neovascularization, and inhibition of fibrosis progression [14]. These findings indicated that MSCs aid in improving the local immune microenvironment of patients, facilitating tissue functional recovery, delaying the progression of chronic diseases, and accomplishing the therapeutic objective of tissue regeneration.

Stem cell therapy employing MSCs has exhibited favorable efficacy in treating diverse diseases [15]. However, due to heterogeneity among patients, this therapeutic effect is quietly different. Thus, identifying, before or during the treatment, patients who are likely or unlikely to respond to stem cell therapy early on is critical in attaining the best therapeutic effects. With the emergence of high-throughput technologies, PBMC is now suitable for deeper immune analysis and serving as crucial biomarkers.

The widespread implementation of single-cell RNA sequencing (scRNA-seq) technology enables researchers to gain a more comprehensive comprehension of the dynamic alterations in peripheral blood mononuclear cells (PBMCs) of heart failure (HF) patients following the administration of stem cell therapy [16]. This study aims to investigate the changes in PBMCs of HF patients with the MSC therapy and determine whether these changes are associated with treatment response. The study comprised 4 HF patients, in which scRNA-seq analysis was performed on 8 PBMC samples before and after cell transplantation. We discovered a significant decrease in the proportion of CD14^+^ monocytes after MSC treatment, suggesting that CD14^+^ monocytes may be the cell type most affected during therapy. Further analysis unveiled subgroups of the therapy-related CD14^+^ monocytes, providing clues to understanding the mechanisms of treatment response and give insights for finding patients suitable for MSC therapies.

## MATERIALS AND METHODS

### Sample collection and RNA-sequencing

This study recruited patients at Shanghai East Hospital (Shanghai, China). The research was conducted in accordance with the ethical principles of the Helsinki Declaration [17] and obtained ethical approval from the Ethics Committee of Shanghai East Hospital. Prior to collection of specimens and clinical information, informed consent was obtained from all registered patients. All patients approved the release of personally identifiable information, received verbal and written project information, and signed a written consent form.

Human umbilical cord mesenchymal stem cells (hUC-MSCs) were administered via intravenous injection at a dose of 1×10^6^ cells/kg for heart failure patients [10]. Clinical assessments and evaluations were conducted on patients one week before cell implantation and at 1 month and 6 months after transplantation. These assessments included routine physical examinations, vital sign monitoring, adverse event evaluation, laboratory tests, and functional measurements (Supplementary Tables 1, 2). The above clinical monitoring showed improvement in patients after stem cell therapy. To further investigate the reasons behind the differences in treatment efficacy among patients, single-cell RNA-sequencing was performed on peripheral blood mononuclear cells (PBMCs) collected from four patients at both pre-treatment and one-month post-treatment, resulting in a total of 8 PBMC samples.

### Public data collection

The GSE59867 used in present work, including PBMC samples from heart failure patients [18], were collected from the Gene Expression Omnibus (GEO) database (https://www.ncbi.nlm.nih.gov/geo/query/acc.cgi?acc=GSE59867). PBMC samples from patients (n=111) with ST-segment elevation myocardial infarction (STEMI) and the control group comprised patients (n=46) with a stable coronary artery disease (CAD) and without a history of myocardial infarction.

### scRNA sequencing for PBMCs from heart failure patients with MSC therapy

The scRNA-seq library was generated using the Chromium Single Cell assay (10× Genomics). The library was sequenced using the NovaSeq 6000 Illumina platform and the raw data were aligned to the human genome (hg38). Quality control and normalization of the scRNA-seq data were performed using the Seurat R package (v4.1.1). The gene expression matrix was normalized to the total UMI counts per cell derived from the filtered cells. We used the FindVariableFeatures function to identify the top 3000 highly variable genes. The FindAllMarkers function in Seurat was executed with default parameters to identify genes exhibiting cluster-specific expression. The cell types of PBMC were annotated based on the WNN algorithm [19]. To determine the composition of samples based on cell types, the cell count for each cell type was calculated from each sample. The aforementioned identical functions were utilized to obtain subclusters of CD14^+^ monocytes for clustering and grouping.

### Metacell analysis of scRNA-seq data

Single-cell transcriptomic data are characterized by high background noise, low gene detection rate, and sparse expression matrix. Currently, it is known that functionally similar genes tend to cluster together, thus algorithmically obtaining metacells often better reflect the true expression profile of cells [20, 21]. Therefore, metacell analysis was conducted on single-cell RNA sequencing data of PBMCs before and after MSC therapy for heart failure, thus providing dependable cellular data for subsequent machine learning modeling.

### Construction of a machine learning model for therapy-response CD14^+^ monocytes

Employing machine learning and leveraging single-cell RNA sequencing to identify crucial regulatory genes may potentially assist in screening for subgroups more suitable for stem cell therapy. The Least Absolute Shrinkage and Selection Operator (LASSO) analysis was utilized to obtain the core genes, and seven machine learning algorithms were employed, resulting in appropriate algorithms and feature genes to better characterize the state of therapy-response CD14^+^ monocytes. The machine learning algorithms were conducted using the mlr3verse package (version 0.2.7) (https://CRAN.R-project.org/package=mlr3verse).

### Gene set enrichment analysis (GSEA) and function enrichment analyses

Gene set enrichment analysis (GSEA) can score sorted gene lists (usually based on fold-change) and calculate if a certain gene set is significantly enriched by permutation test [22]. We used GSEA to investigate the potential role of response-related monocytes in the process of MSCs treating heart failure. In parallel, the Gene Ontology and KEGG enrichment analyses were also performed using clusterProfiler package [23].

### Trajectory analysis for CD14^+^ monocytes

The Monocle 2 algorithm can elucidate the changing trends of gene expression as cellular states transition, even revealing hidden patterns of variation [24]. We employed pseudo-time analysis to investigate the process of patient response to MSC treatment before and after, and depicted the expression profiles of 7 key genes that change over time.

### Single-cell metabolism analysis

The scMetabolism package was used to calculate the single-cell metabolic activity of cell subpopulations. In particular, the VISION algorithm was adopted and the Kyoto Encyclopedia of Genes and Genomes (KEGG) metabolic gene set was employed for analysis [25]. The DotPlot.metabolism function is used for visualization.

### SCENIC analysis for CD14^+^ monocytes

Single-cell regulatory network inference and clustering (SCENIC) is a GRN (gene regulatory network) algorithm specifically developed for single-cell data [26]. Its innovation stems from the incorporation of a gene co-expression network, deduced through transcription factor motif sequence validation statistical methods, which are capable of identifying highly reliable, TF-driven GRNs. Using SCENIC analysis, we further emphasized the importance of FOS.

### Statistical analysis

Statistically significant difference is calculated in R. *P*-values of less than 0.05, 0.01 and 0.001 were considered statistically significant.

### Data and code availability

The data supporting the research findings can be found in the main text and supplementary manuscript. Other data could be obtained by contacting the corresponding author.

### Consent for publication

The authors agree with the terms of conditions for publication.

## RESULTS

### Characteristics of PBMCs before and after MSC therapy for HF patients

**Figure 1:**
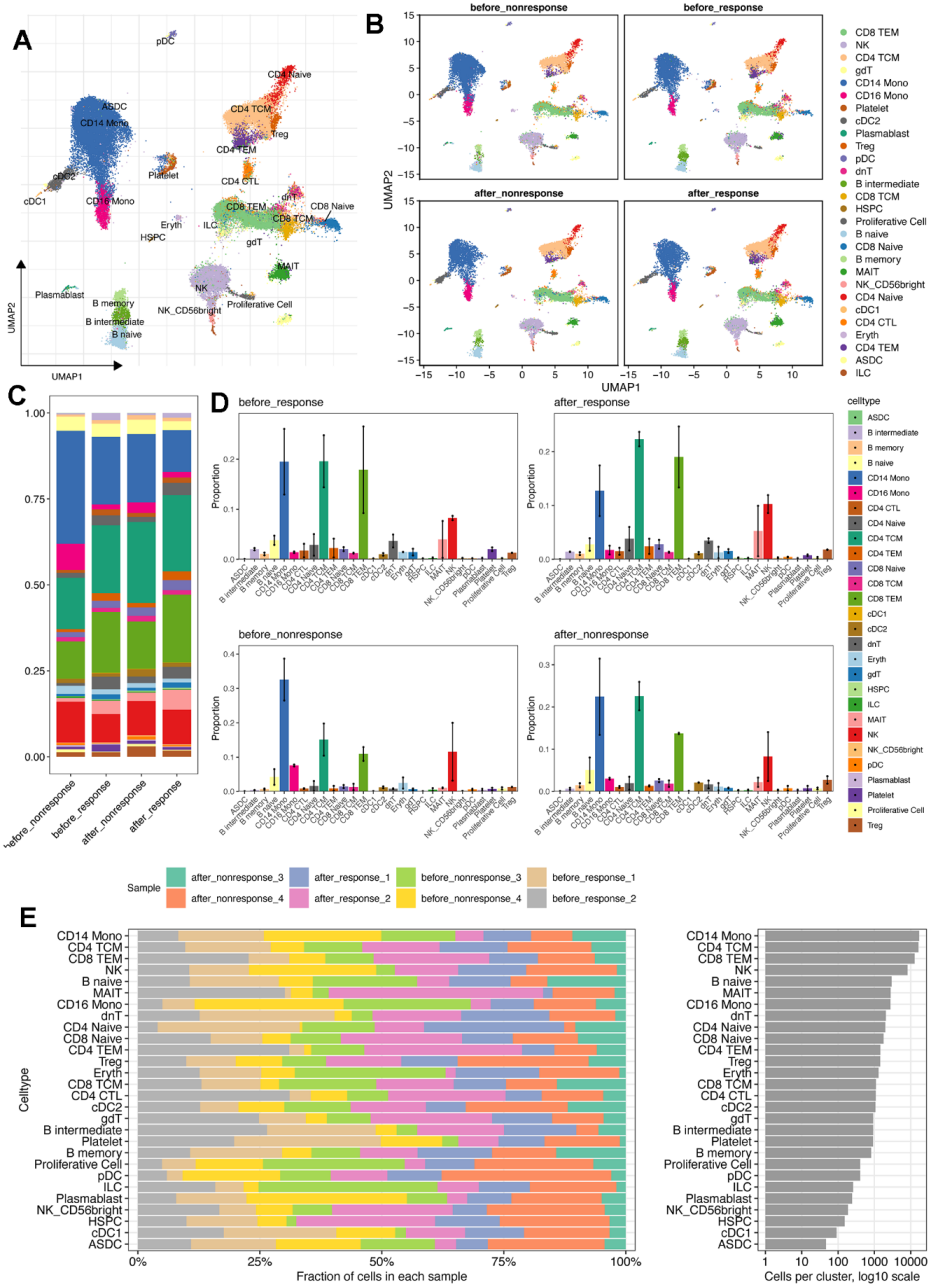
**scRNA-seq reveals the different cell types and distribution among patients with or without MSCs treatment.** (**A**) Cell annotation using the azimuth package (https://app.azimuth.hubmapconsortium.org/app/human-pbmc). And the referenced UMAP was leveraged to better visualized the results. (**B**) Cell distribution for all four groups. (**C**) Cell proportion of all cell types in four groups. (**D**) Bar plot shows the cell proportion of all cell types in four groups. (**E**) Cell distribution analysis indicates the largest cell proportion of CD14^+^ monocytes.

To better understand the circulating system changes of heart failure patients before and after stem cell therapy, we conducted scRNA-seq for bettering depicting the expression pattern of peripheral blood mononuclear cells (PBMCs) of HF patients. As showed in Figure 1A and Supplementary Tables 1, 2, we collected four patients who have received MSC therapy (Supplementary Tables 1, 2). As a result, there were 81226 single cells obtained after stringent criteria (Supplementary Figures 1, 2). As shown in Figure 1A, 1B, the detected single cells were annotated into different cell types using “Weighted nearest neighbor” analysis and the referenced UMAP plot from the multimodal PBMC reference was adopted for better visualizing the scRNA-seq data [19]. Furthermore, we tried to explore the expression characteristics of different cell types, especially changes of cell proportion and cell count. Intriguingly, the proportion of CD14^+^ monocytes was down-regulated after MSC therapy, no matter in the response group or non-response group (Figure 1C, 1D). Moreover, the total cell number of CD14^+^ monocytes (17048 cells) was more than other cell types (Figure 1E). Then, the metabolism analysis of CD14^+^ monocytes was performed and results showed that many metabolism pathways varied among four groups (Supplementary Figure 3), In brief, CD14^+^ monocytes might be affected most with MSCs therapy.

### Nonnegative matrix factorization (NMF) analysis showed the heterogeneity of response and non-response CD14^+^ monocytes

To compare the different of CD14^+^ monocytes before and after MSC therapy, we utilized three methods to obtain conserved markers of CD14^+^ monocytes in different groups: (i) method 1: the “findallmarker” function in Seurat package [19] was used, which would compare the interested cell cluster to other all cells using Wilcox algorithm; (ii) method 2: the “findmarker” function Seurat package was adopted, and then we compared the interested cell cluster one by one using Wilcox algorithm. At last, the intersected marker genes were considered specific to the interested cell cluster; (iii) method 3: Metacell algorithm was applied and we obtained the “de-noised” metacells [20, 21] and thereafter we could use the DESeq2 package [27] for differential expression analysis. With these three methods, we could obtain the highly conserved markers for the four group of CD14^+^ monocytes.

**Figure 2:**
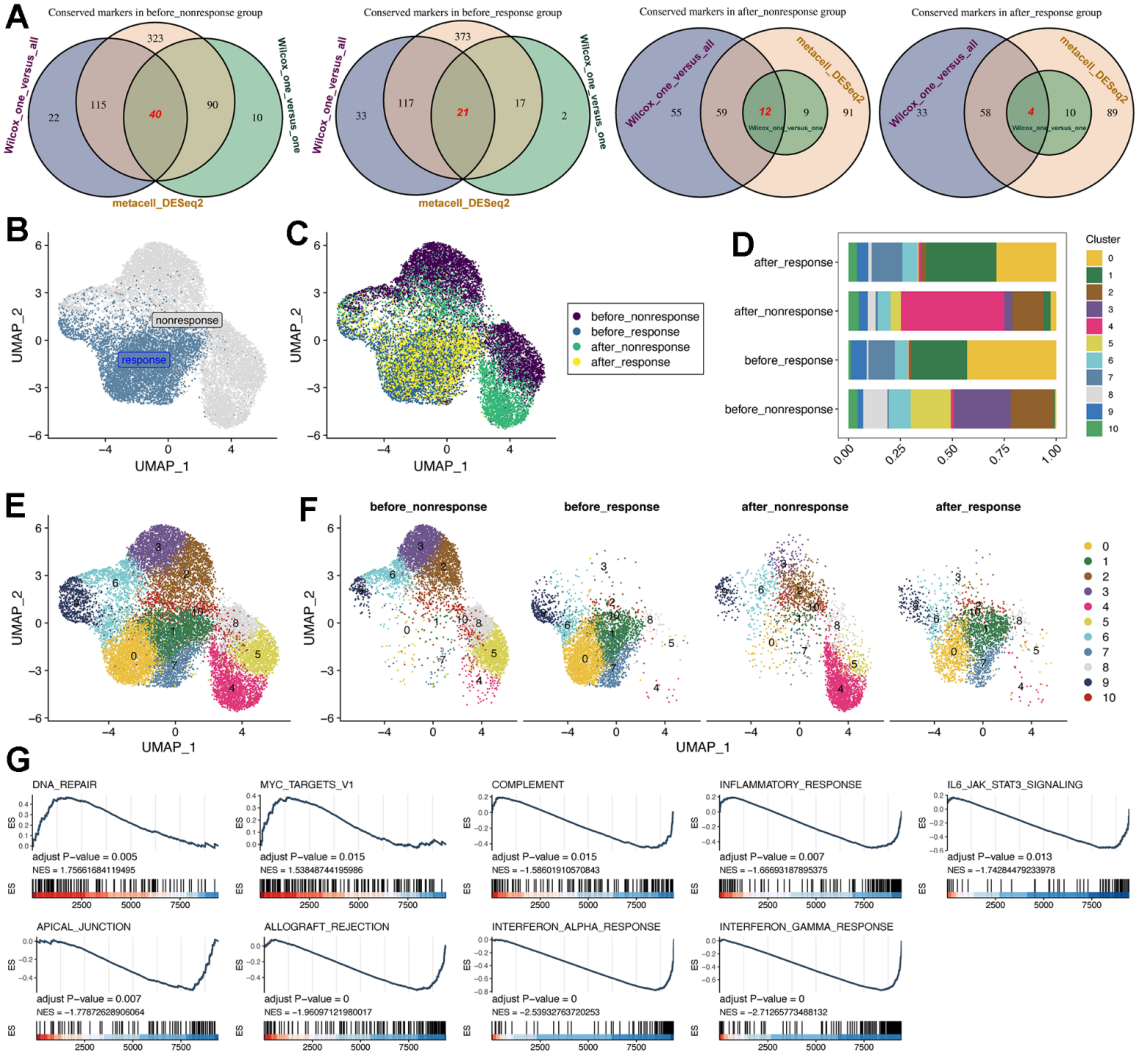
**Subcluster analysis of the CD14^+^ monocytes.** (**A**) Identification of highly-conserved DEGs/markers for CD14^+^ monocytes in the four groups, respectively. Three approaches for exploring DEGs were adopted. DEGs: differentially expressed genes. (**B**) NMF analysis for the reduction analysis of CD14^+^ monocytes based on all highly-conserved DEGs/markers. NMF: Nonnegative Matrix Factorization. (**C**) Distribution of all CD14^+^ monocytes from four groups. (**D**) Cell proportion of CD14^+^ monocytes was visualized, comprising 11 subclusters. (**E**) Cell distribution of all 11 identified subclusters. (**F**) Cell distribution of all 11 identified subclusters in the four groups. The CD14^+^ monocytes from response and non-response patients were quite different. (**G**) GSEA pipeline for showing the enriched terms for CD14^+^ monocytes from the “before response” group.

Subsequently, with the highly conserved markers, we run the NMF analysis for understanding the heterogeneity and potential functions of CD14^+^ monocytes (Figure 2B, 2C). Of interest, the response and non-response groups were obviously separated using the UMAP plot (Figure 2B), indicating the difference of CD14^+^ monocytes in response and non-response group. However, the CD14^+^ monocytes before and after MSC therapies in the response group were quite similar while relatively distinct in the non-response group (Figure 2C), demonstrating that CD14^+^ monocytes might be capable of serving as important indicators of whether and which HF patients might be suitable for MSC therapy or could be benefit from MSC therapy.

For better investigating the characteristics of CD14^+^ monocytes in the “before_response” group, we utilized the subcluster analysis based on the NMF dimension reduction. As a result, 11 subclusters of CD14^+^ monocytes were obtained (Figure 2D–2F). Then, we used GO (Supplementary Figure 4), KEGG and Reactome (Supplementary Figure 5) databases for function enrichment analyses. Interestingly, we confirmed the various function of different subclusters. For instance, the cluster 0 was enriched in “IL−17 signaling pathway”, “Neutrophil degranulation” and “cellular response to chemical stress”, while cluster 1 was enriched in “Viral myocarditis”, “Interferon Signaling” and “positive regulation of leukocyte cell− cell adhesion”. Then, we also found that cluster 0 (2786 cells) was predominantly enriched in the “before_response” group and would be down-regulated after MSC treatment (Figure 2F). Thus, we identified cluster 0 as “therapy-response CD14^+^ monocytes”. Further, we leveraged the GSEA analysis for running the enrichment analysis of HALLMARK datasets (downloaded from http://www.gsea-msigdb.org). Of interest, the potential function of response CD14^+^ monocytes were mainly related to “COMPLEMENT”, “INFLAMMATORY RESPONSE”, “INTERFERON_GAMMA_RESPONSE” and so on, which were highly related to the therapeutic mechanism of MSCs (Figure 2G).

### Machine learning algorithms were leveraged to construct the predictive model for therapy-response CD14^+^ monocytes and identified potential indicators

**Figure 3:**
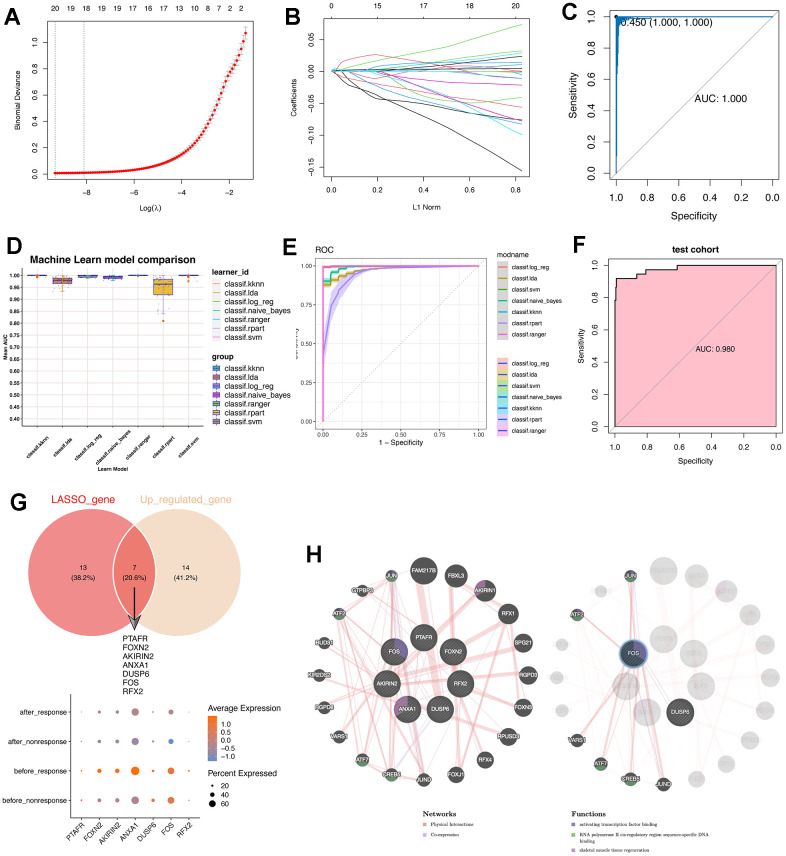
**Machine learning algorithms were leveraged to construct a predictive model for therapy-response CD14^+^ monocytes and explored potential indicators.** (**A**) LASSO algorithm was adopted for filtering optimal feature genes. Only the 21 highly-conserved makers/DEGs for therapy-response CD14^+^ monocytes were adopted. (**B**) Coefficients of identified feature genes from LASSO algorithm was shown. (**C**) ROC analysis for the LASSO model. LASSO: Least absolute shrinkage and selection operator. (**D**) Machine learning algorithm for constructing a predictive model for therapy-response CD14^+^ monocytes. (**E**) ROC analysis for the machine learning model. (**F**) ROC analysis for the machine learning model in test cohort. The AUC value was 0.98. AUC: area under curve. (**G**) Intersection of genes identified by the LASSO algorithm with the highly conserved markers of the therapy-response CD14^+^ monocytes. Seven overlapped genes were delineated. (**H**) Protein-protein interaction network for identified genes. The network prediction was based upon an online web-server: GeneMANIA (http://www.genemania.org).

To identify the therapy-response CD14^+^ monocytes in single cell levels, we used the metacell algorithm [20, 21] and divided CD14^+^ monocytes into training cohort and test cohort. Using LASSO algorithm, we had filtered the 77 conserved markers and only 20 features were left (Figure 3A, 3B), including IFI6, PTAFR, S100A12, FOXN2, MAP3K1, CAST, F13A1, HLA-DPB1, AKIRIN2, ANXA1, DUSP6, EPSTI1, FOS, SOCS3, RFX2, TPM4, ZFP36, PPDPF, MX2 and RPL39. Then, we used the “Bootstrap” algorithm for internal validation for 1000 times and the performance was reliable (Figure 3C). To avoid the bias effect of different algorithms, we further used another seven machine algorithms for constructing the classifier for therapy-response CD14^+^ monocytes (Figure 3D). Intriguingly, the performance of all seven machine algorithms was quite robust, especially SVM algorithm (Figure 3E). Then, we used the test cohort for further validation, similar result was obtained that the performance of the constructed classifier was good (AUC: 0.98) (Figure 3F).

Then we also intersected the machine learning-identified feature genes and the conserved up-regulated genes in the “before_response” group (Figure 3G). Result showed that PTAFR, FOXN2, AKIRIN2, ANXA1, DUSP6, FOS and RFX2 were obtained (Figure 3G and Supplementary Figure 6). Then, we used the GeneMANIA for constructing real-time multiple association network integration [28] (Figure 3H). Of note, the FOS/JUN-related transcription factors were enriched.

### Trajectory analysis of therapy-response CD14^+^ monocytes

**Figure 4:**
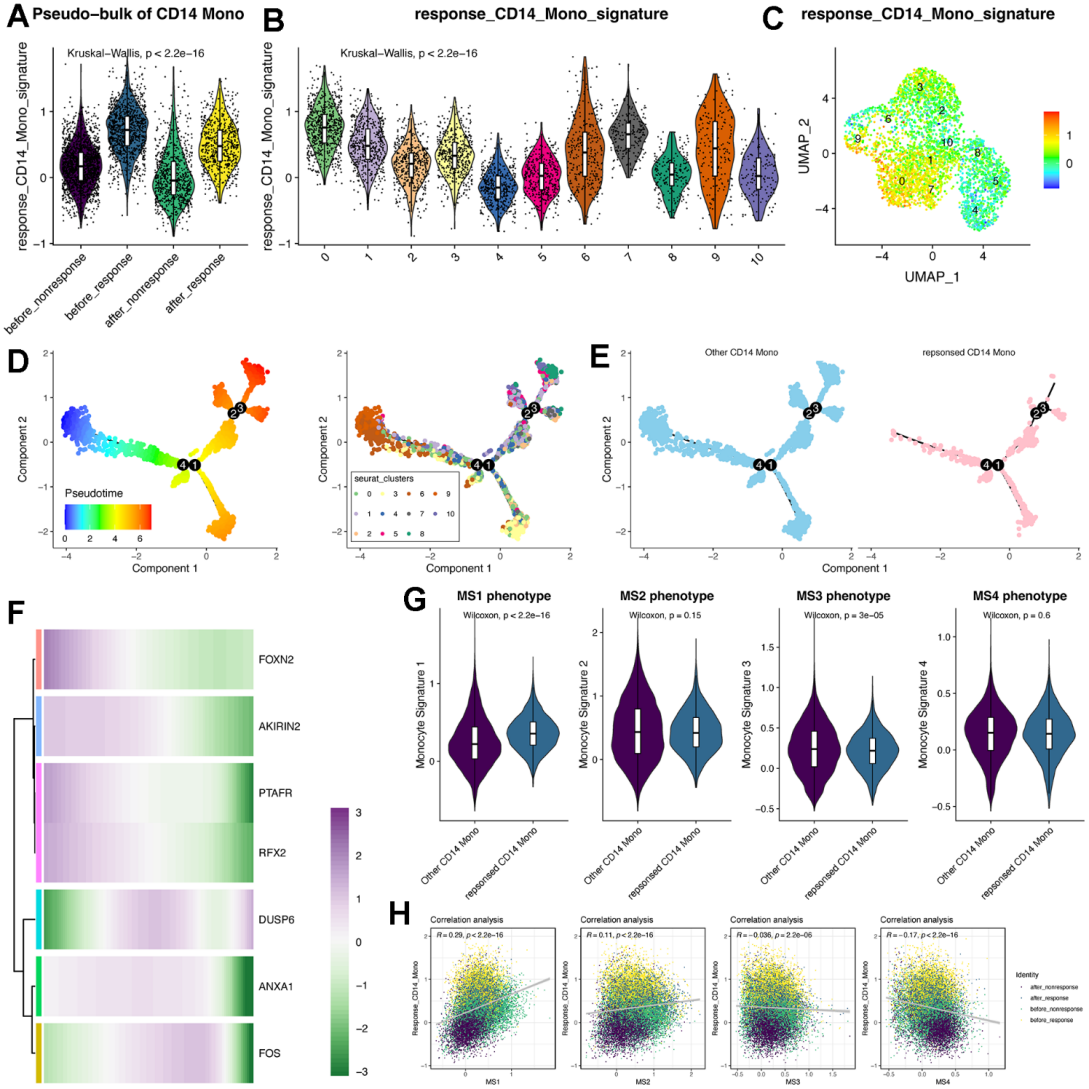
**Trajectory analysis of CD14^+^ monocytes and the pseudotime changes of the feature genes identified by machine learning algorithms.** (**A**) Violin plot displays the response-related CD14^+^ monocyte signature in pseudo-bulk levels of CD14^+^ monocytes. (**B**) Violin plot displays the response-related CD14^+^ monocyte signature across all identified subclusters of CD14^+^ monocytes. “AddModule” function in Seurat package was leveraged to estimate the signature score. (**C**) Signature score of response-related CD14^+^ monocytes was mainly activated in cluster 0. (**D**) Trajectory analysis of CD14^+^ monocytes. Monocle2 package was adopted. (**E**) Pseudotime distribution of other and response-related CD14^+^ monocytes. (**F**) Expression changes of the feature genes following pseudotime. (**G**) Violin plot shows the monocyte signature scores. MS: monocyte signature. (**H**) Correlation analysis between the response signature and the other four published MS phenotypes.

Then, we used the “addmodule” function in Seurat package for calculating the signature score of the seven therapy-response-specific and up-regulated genes (Figure 4A–4C). Of note, the signature score was higher in CD14^+^ monocytes from the “before_response” group (Figure 4A) and was also higher in cluster 0 (therapy-response CD14^+^ monocytes) (Figure 4B, 4C). Then, we performed the pseudotime analysis using monocle2 algorithm [24]. Intriguingly, we could find that the cluster 0 was mainly distributed in the early and middle timepoint of the pseudotime plot (Figure 4D, 4E). Then, we detected the expression pattern of the seven genes, most of which were consistent with the pseudotime distribution (Figure 4F). Considering that the middle stage character and potential FOS/JUN-related function of therapy-response CD14^+^ Monocytes, which indicated that the therapy-response CD14^+^ Monocytes might be much more flexible and its state would be shifted, it triggered us to understand whether its status might be similar to the published monocyte signature (MS) reported by Reyes et al. [29], whose research has found four monocyte signatures in the PBMC of sepsis patients and considered MS1 as a unique disease-related CD14^+^ monocytes state that is expanded in sepsis patients and has validated its power in distinguishing sepsis patients from healthy controls [29]. Interestingly, we found that the therapy-response CD14^+^ monocytes also had higher MS1 score than other CD14^+^ monocytes (Figure 4G) as well as positively correlated with the MS1 score (Figure 4H). In summary, the state of therapy-response CD14^+^ monocytes might also be different from other CD14^+^ monocytes, and might be an indicator of those HF patients with could potentially benefit from MSC therapy.

### SCENIC analysis revealed potential regulators for therapy-response CD14^+^ monocytes

**Figure 5:**
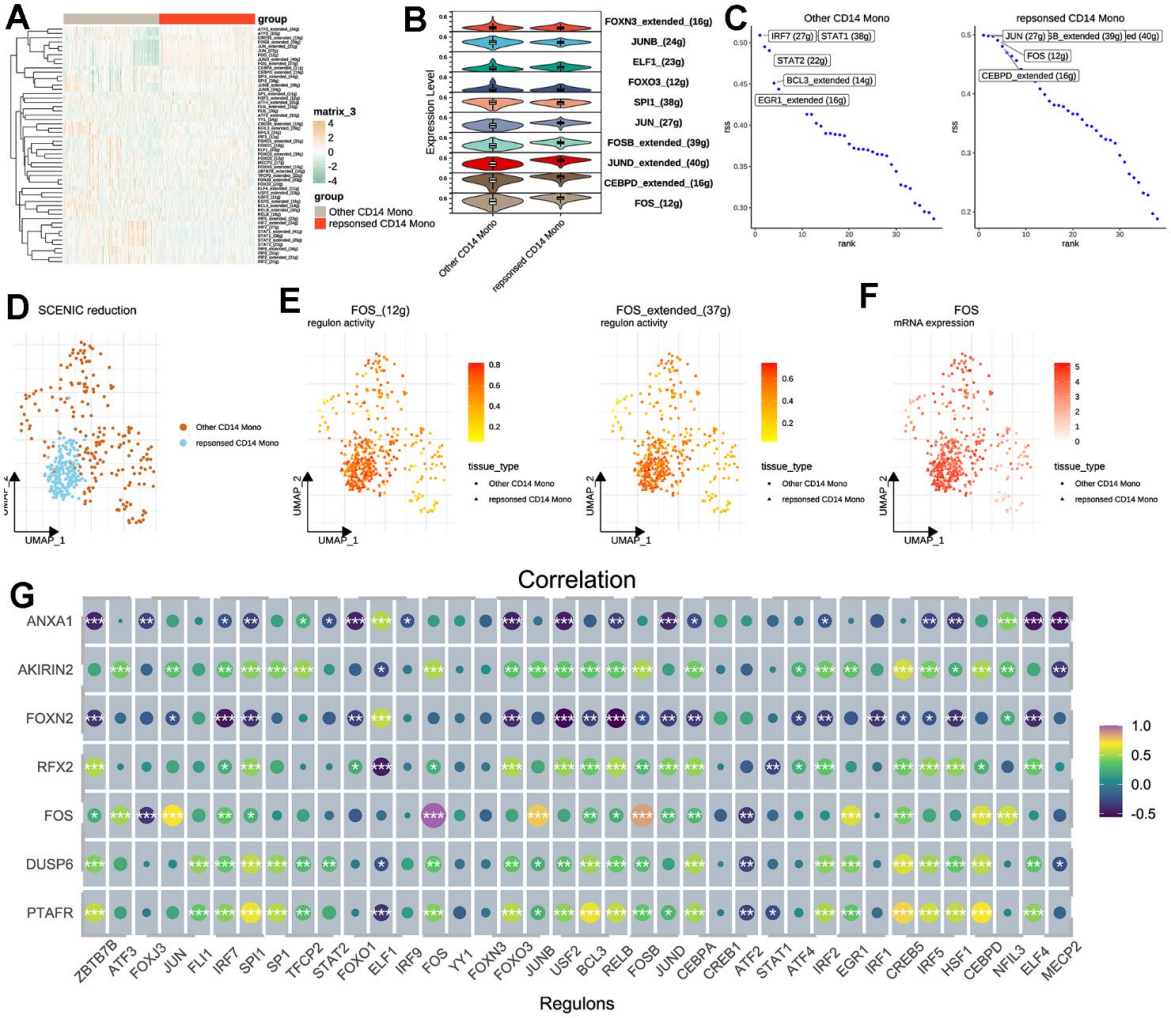
**Transcription factor analysis for CD14^+^ monocytes.** (**A**) SCENIC pipeline for identifying potential regulatory TFs for therapy-response CD14^+^ monocytes and heat map was used for visualization. SCENIC: Single-Cell rEgulatory Network Inference and Clustering; TF: Transcription factor. (**B**) Violin plot for showing the identified TFs between other and therapy-response CD14^+^ monocytes. (**C**) Ranking of the potential TFs for other and therapy-response CD14^+^ monocytes. (**D**) Cell distribution based on the identified regulons/TFs. (**E**) Activities of FOS regulons in UMAP plots. Two regulons of FOS were considered significant. (**F**) Expression levels of FOS shown by UMAP plot. (**G**) Correlation analyses among identified TFs and hub feature genes.

Then, we tried to explore the transcriptional regulatory network of CD14^+^ monocytes. As a result, the activities of several TFs were different between therapy-response CD14^+^ monocytes and other CD14^+^ monocytes (Figure 5A). The top TFs were showed in Figure 5B, 5C. Data showed that therapy-response CD14^+^ monocytes were mainly regulated by JUN, FOS, CEBPD while other CD14^+^ monocytes were predominantly controlled by IRF7, STAT1 and BCL3 (Figure 5C). More than this, therapy-response CD14^+^ monocytes could be aggregated in the UMAP plot based on regulon activity scores calculated by SCENIC (Figure 5D). Noteworthily, the FOS regulons (FOS_extend_(37g) and FOS_(12g)) and FOS mRNA expression level were all highly up-regulated in therapy-response CD14^+^ monocytes (Figure 5E, 5F), underpinning the crucial roles of FOS in regulating the functions of therapy-response CD14^+^ monocyte signature. Taking it a step further, we downloaded published sequencing data (GSE59867) of PBMC related to post-MI HF patients and result showed significant correlations between detected TFs and the seven genes (Figure 5G). To further understand the role of FOS, we also predicted the potential drugs for both capable of activating FOS and also treating heart diseases (Supplementary Table 3). Patients who have adopted these drugs for HF therapy might be much suitable for MSC therapy.

### NMF subtype analyses reveals monocyte signatures were capable of classifying post-MI HF patients into three groups

**Figure 6:**
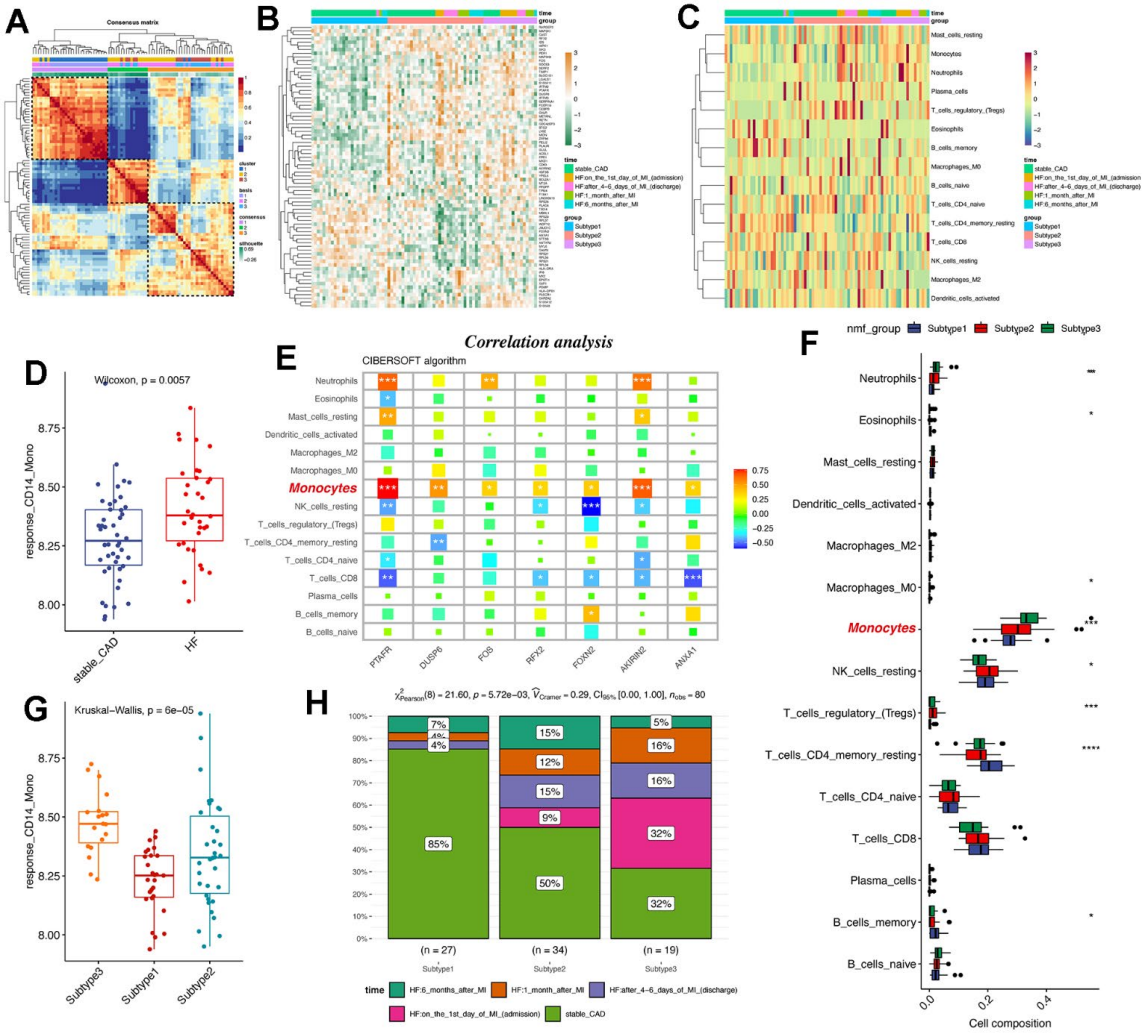
**NMF subtype analyses uncovers that therapy-response monocyte signatures were capable of partitioning post-MI HF patients into three groups.** (**A**) Consensus clustering for the GSE59867 data based on the hub genes, which contained PBMC samples from stable coronary diseases to heart failure. (**B**) Heatmap shows the differences among the three patient groups. (**C**) Heat map displays the infiltration levels of immune cells among the three patient groups. CIBERSOFT algorithm in IOBR packages was adopted. (**D**) Boxplot shows the signature score of therapy-response monocytes in bulk RNA-seq levels between control and heart failure groups. (**E**) Correlation analysis of the hub genes and monocytes in bulk RNA-seq levels. The monocytes were highlighted. (**F**) Infiltration levels of deconvoluted immune cells in the NMF groups. (**G**) Box plot reveals the signature score of therapy-response monocytes among the three patient groups. (**H**) Distribution of different clinical information in the NMF groups. NMF: Nonnegative Matrix Factorization.

Considering the pivotal roles of monocytes in post-MI heart failure, we tried to further discover whether the conserved markers we identified were powerful enough to distinguish HF patients from healthy patients. Herein, we also used the GSE59867 data, which contained PBMC samples from stable coronary diseases to heart failure. Using NMF analysis [30] (Supplementary Figure 7), we had classified stable CAD patients and post-MI patients into three subgroups based on the 77 conserved markers of CD14^+^ monocytes (Figure 6A, 6B). Interestingly, FOS was highly expressed in subgroup 3 (Figure 6B). Then, we used CIBERSOFT algorithm in IOBR packages [31] to deconvolute the bulk sequencing data for understanding the immune filtration characters (Figure 6C) response CD14^+^ monocyte signature and the signature score was higher in HF patients compared to stable CAD patients (Figure 6D). Correlation analysis reveals that CIBERSOFT-inferred monocytes were positively correlated with all seven therapy-response-related genes (Figure 6E) and monocytes were also highly expressed in subgroup 3 (Figure 6C, 6F) compared to subgroup 1 and 2. Consistently, the therapy-response CD14^+^ monocyte signature was also highly expressed in subgroup 3 (Figure 6G). Finally, the distribution of patients was showed (Figure 6H). In brief, CD14^+^ monocyte participated in the development of post-MI HF patients and the therapy-response CD14^+^ monocyte signature might be considered as typical changes of subgroup 3.

## DISCUSSION

Heart failure represents a significant global health burden, with increasing prevalence and mortality rates worldwide. Despite significant advancements in medical therapies, the prognosis for HF patients remains poor, with limited options for complete cardiac functional restoration [32]. In recent years, MSC-based therapies have emerged as promising approaches for the treatment of HF [4]. Preclinical studies in animal models and initial clinical trials have provided encouraging results, demonstrating the safety, feasibility, and potential efficacy of stem cell therapy in HF [10–12]. Stem cells have been shown to promote angiogenesis, reduce scar formation, and improve cardiac function through paracrine effects, direct differentiation, and integration into the damaged myocardium [9]. However, MSC therapies are expensive and it is still lacking of enough biomarkers for identifying the suitable HF patients for accepting MSC therapies and therefore enlarging the application and benefits of MSC therapies. Understanding the changes of PBMCs of HF patients responded or not responded to MSC therapies might pave the way for accurate approaches and ultimately improve patient outcomes.

Monocytes exhibit dual effects in acute heart failure, and their relative contributions may vary at different disease stages. In the early stage of acute heart failure, monocytes can migrate to myocardial tissue and serve multiple functions, such as removing inflammatory substances in the heart tissue, reducing apoptosis, pyroptosis, and necrosis of cardiomyocytes, supporting damaged cardiomyocyte repair, promoting angiogenesis, and providing immune protection. However, with the deterioration of heart failure, monocyte-mediated inflammatory response may lead to adverse myocardial remodeling and interstitial fibrosis, resulting in impaired cardiac contractile function. The findings of this study indicate that the proportion of CD14^+^ monocytes in the blood of patients is significantly reduced after MSC therapies, which might be related to post-treatment inflammation or immune reactions [33–36]. Stem cell therapy may elicit an inflammation-mediated response, activate CD14^+^ monocytes, induce their migration to inflammatory sites, and subsequently reduce their presence in peripheral blood. Secondly, MSC therapies may also promote the repair of cardiac tissue, which could also lead to the migration of CD14^+^ monocytes to damaged tissue to support the repair process. Thirdly, stem cell therapy may possess immunosuppressive effects that alleviate inflammation and thereby decrease the number of CD14^+^ monocytes in peripheral blood. Of course, CD14^+^ monocytes may be involved in the inflammatory or repair process and eventually undergo apoptosis or necrosis, further leading to their decrease in peripheral blood [33–36]. Recently, a study also reported that the CCL2^+^DPP4^+^ MSCs can response to CCL20^+^CD14^+^ monocytes and IL-6, and thereby lead to the formation of creeping fat [37], indicating the existence of the crosstalk between MSCs and CD14^+^ monocytes. Taken together, the decrease of CD14^+^ monocytes may be a part of the complex biological response after stem cell therapy. This study did not conduct further experimental researches on the mechanisms for the reduction of CD14^+^ monocytes in peripheral blood, but we found that heterogeneous information on CD14^+^ monocytes may serve as a crucial indicator/biomarker to evaluate whether HF patients are suitable for MSC treatment because different subgroups of CD14^+^ monocytes exhibit functional enrichment in different physiological processes, and some functional enrichment pathways are closely related to the mechanisms of MSC treatment, which helps explain the effectiveness of MSC therapies.

Using machine learning algorithms, we developed a precise prediction model for therapy-related CD14^+^ monocytes and identified seven highly expressed genes that play a crucial role in therapy-related CD14^+^ monocytes. Among these genes, we found those related to the AP-1 family, including JUN and FOS. On the other hand, SCENIC analysis revealed the potential regulatory mechanisms of therapy-related CD14^+^ monocytes, in which transcription factors such as JUN and FOS might play key roles. The AP-1 (Activator Protein-1) family is typically composed of members from the FOS and JUN protein families. AP-1 is a transcription factor that binds to DNA and regulates the expression of multiple genes. The AP-1 complex regulates the expression of many genes by binding to specific regions of DNA [38]. These genes encompass various biological processes, such as cell proliferation, growth, inflammatory response, apoptosis, and cell differentiation [39, 40]. Furthermore, we attempted to classify HF patients from public databases into different subgroups and found that PBMCs of heart failure patients had heterogeneity and could be further divided into three groups. The expression of therapy-related CD14^+^ monocytes in these groups was significantly different, which suggests that the activation of AP-1 family and the therapy-related CD14^+^ monocytes may help identify and screen heart failure patients suitable for the MSCs therapy.

The present study has certain limitations. Firstly, the scRNA-seq data included only 4 HF patients and were divided into two groups based on treatment efficacy, thus the sample size was relatively small. Furthermore, this study mainly focused on the changes and therapeutic responses of CD14^+^ monocytes, while stem cell therapy for heart failure involves complex cell interactions. In addition, the construction of the machine learning model was a highlight of this study but requires further validation. The stability and reliability of the model need to be validated in larger independent sample sets to determine its effectiveness in clinical applications. More importantly, this study chose the time point of 1 week after MSC treatment, but HF patients might benefit over a longer time range after the MSC therapy. Further research could consider samples from different time points to better understand the evolution of treatment effects and screen for more effective evaluation biomarkers.

## CONCLUSIONS

Through single-cell RNA sequencing, we identified a subpopulation of treatment-associated CD14^+^ monocytes. In addition, SCENIC analysis revealed the potential regulatory mechanisms of treatment-responsive CD14^+^ monocytes, highlighting the critical role of transcription factors of the AP-1 family, such as JUN and FOS. The machine learning model based on CD14^+^ monocytes can effectively predict CD14^+^ monocytes. Seven genes highly expressed in treatment-responsive CD14^+^ monocytes can be used for subtyping analysis of heart failure patients.

## Supplementary Material

Supplementary Figures

Supplementary Tables
