# Supplementary material for: Single cell RNA-seq identifies a FOS/JUN-related monocyte signature associated with clinical response of heart failure patients with mesenchymal stem cell therapy
Source: Aging (Albany NY). 2024 Mar 20;16(6):5651–75. doi: 10.18632/aging.205670 (PMC11006470; doi:10.18632/aging.205670)
Supplement: Supplementary Figures [file aging-16-205670-s001.pdf]

SUPPLEMENTARY FIGURES

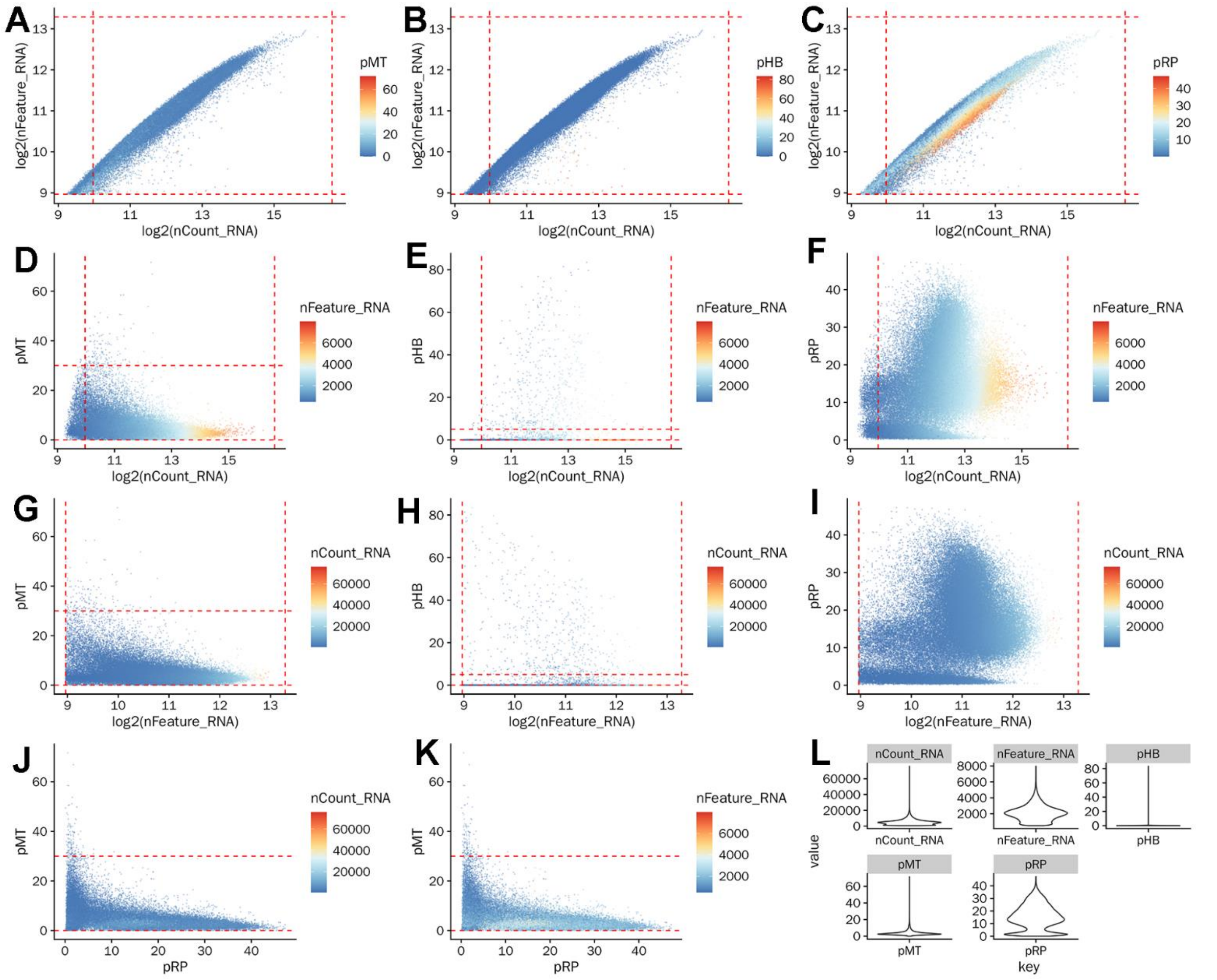

Supplementary Figure 1. Quality of the single-cell RNA-seq data of heart failure patients. Scatter plot shows the relationships between nCount_RNA and nFeature_RNA. Color changes represent the changes of pMT (**A**), pHB (**B**), and pRP (**C**). Scatter plot shows the relationships between nCount_RNA and pMT (**D**), pHB (**E**) and pRP (**F**). Color changes represent the changes of nFeature_RNA. Scatter plot displays the relationships between nFeature_RNA and pMT (**G**), pHB (**H**) and pRP (**I**). Color changes represent the changes of nFeature_RNA. Scatter plot shows the relationships between pRP and pMT. Color changes represent the changes of nCount_RNA (**J**) and nFeature_RNA (**K**). (**L**) Violin plot displays the values of total RNA read count (nCount_RNA), total gene count (nFeature_RNA), pHB (expression percentage of hemoglobin genes), pMT (expression percentage of mitochondria genes) and pRP (expression percentage of ribosome protein genes).

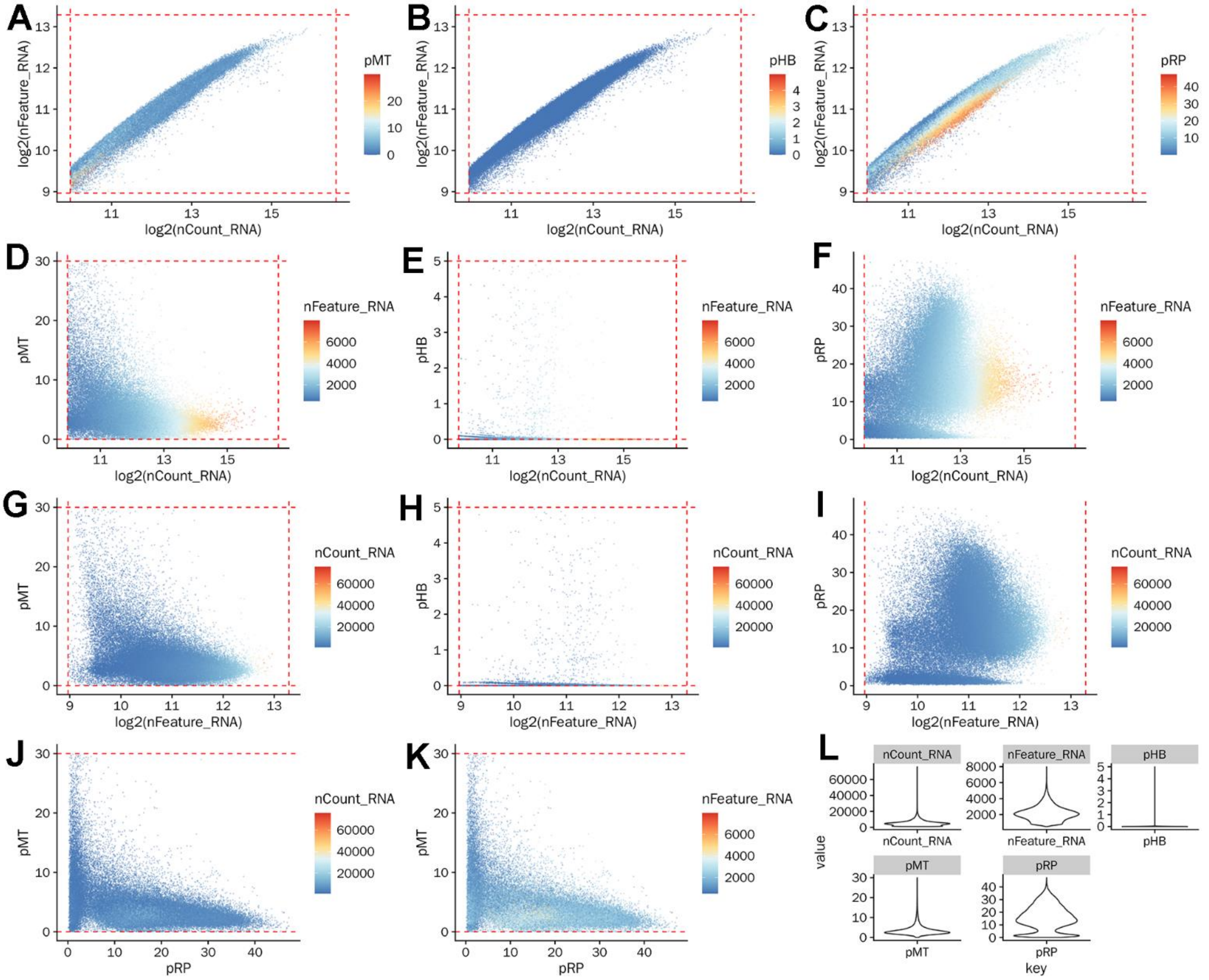

Supplementary Figure 2. Quality control of the single-cell RNA-seq data of heart failure patients with stringent criteria. Scatter plot shows the relationships between nCount_RNA and nFeature_RNA. Color changes represent the changes of pMT (**A**), pHB (**B**), and pRP (**C**). Scatter plot shows the relationships between nCount_RNA and pMT (**D**), pHB (**E**) and pRP (**F**). Color changes represent the changes of nFeature_RNA. Scatter plot displays the relationships between nFeature_RNA and pMT (**G**), pHB (**H**) and pRP (**I**). Color changes represent the changes of nFeature_RNA. Scatter plot shows the relationships between pRP and pMT. Color changes represent the changes of nCount_RNA (**J**) and nFeature_RNA (**K**). (**L**) Violin plot displays the values of total RNA read count (nCount_RNA), total gene count (nFeature_RNA), pHB (expression percentage of hemoglobin genes), pMT (expression percentage of mitochondria genes) and pRP (expression percentage of ribosome protein genes).

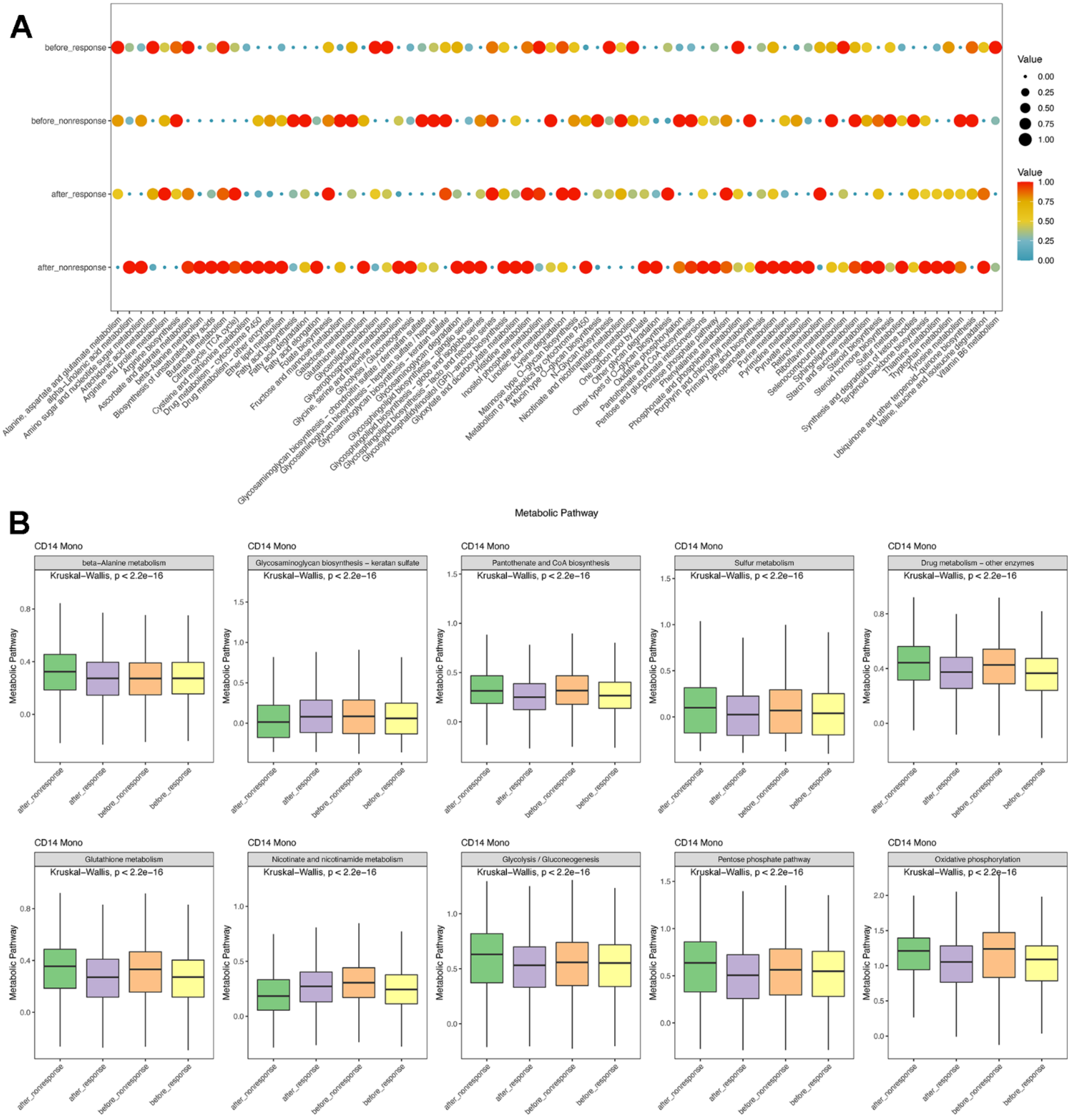

Supplementary Figure 3. Single-cell metabolism analysis for CD14^{+} monocytes in the four groups. (**A**) Dot plot shows all metabolism terms among response or non-response groups. (**B**) Top 10 metabolism terms were shown using box plots.

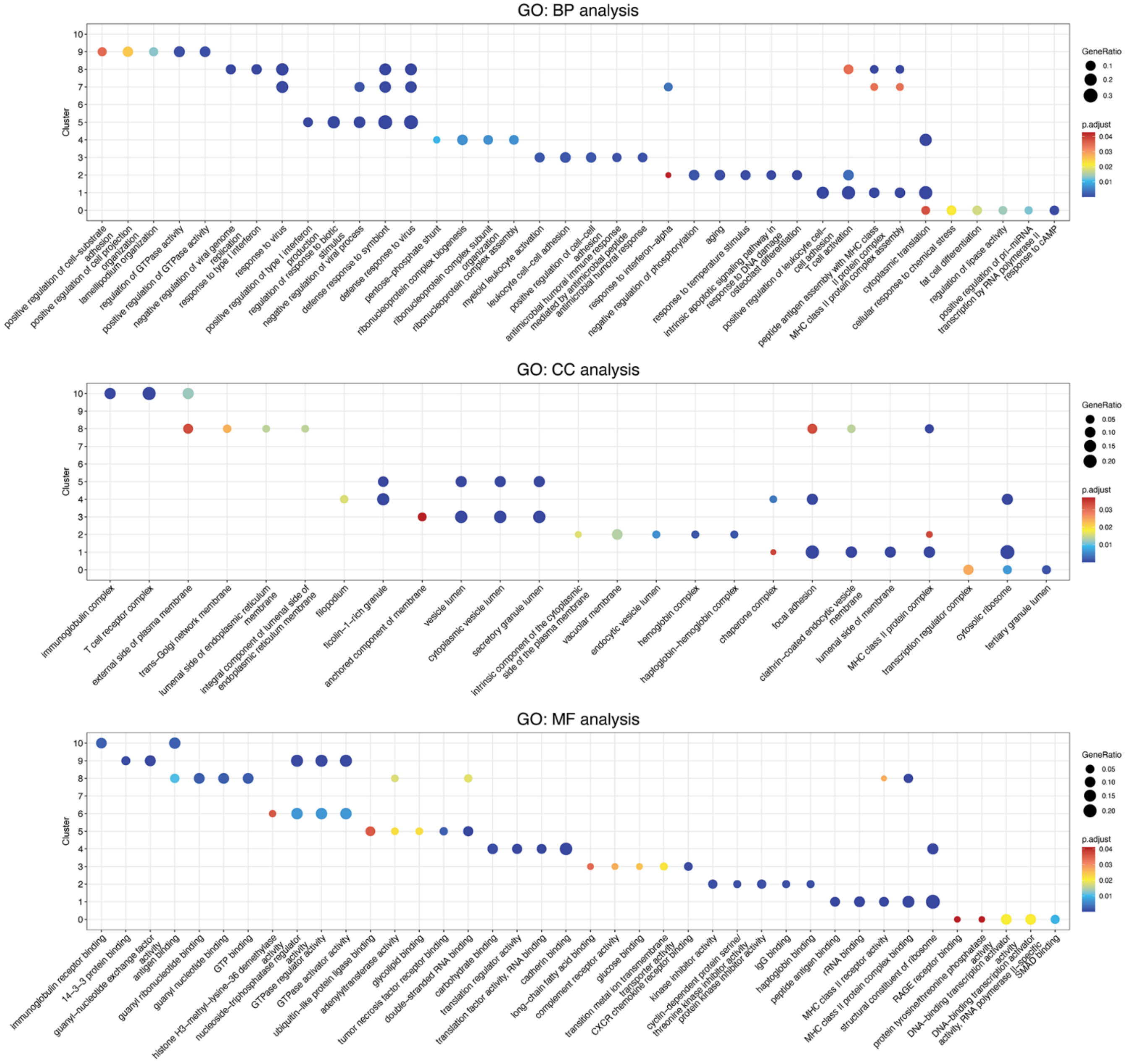

Supplementary Figure 4. GO enrichment analysis for all 11 subclusters of CD14$^+$ monocytes. Dot plots were used.

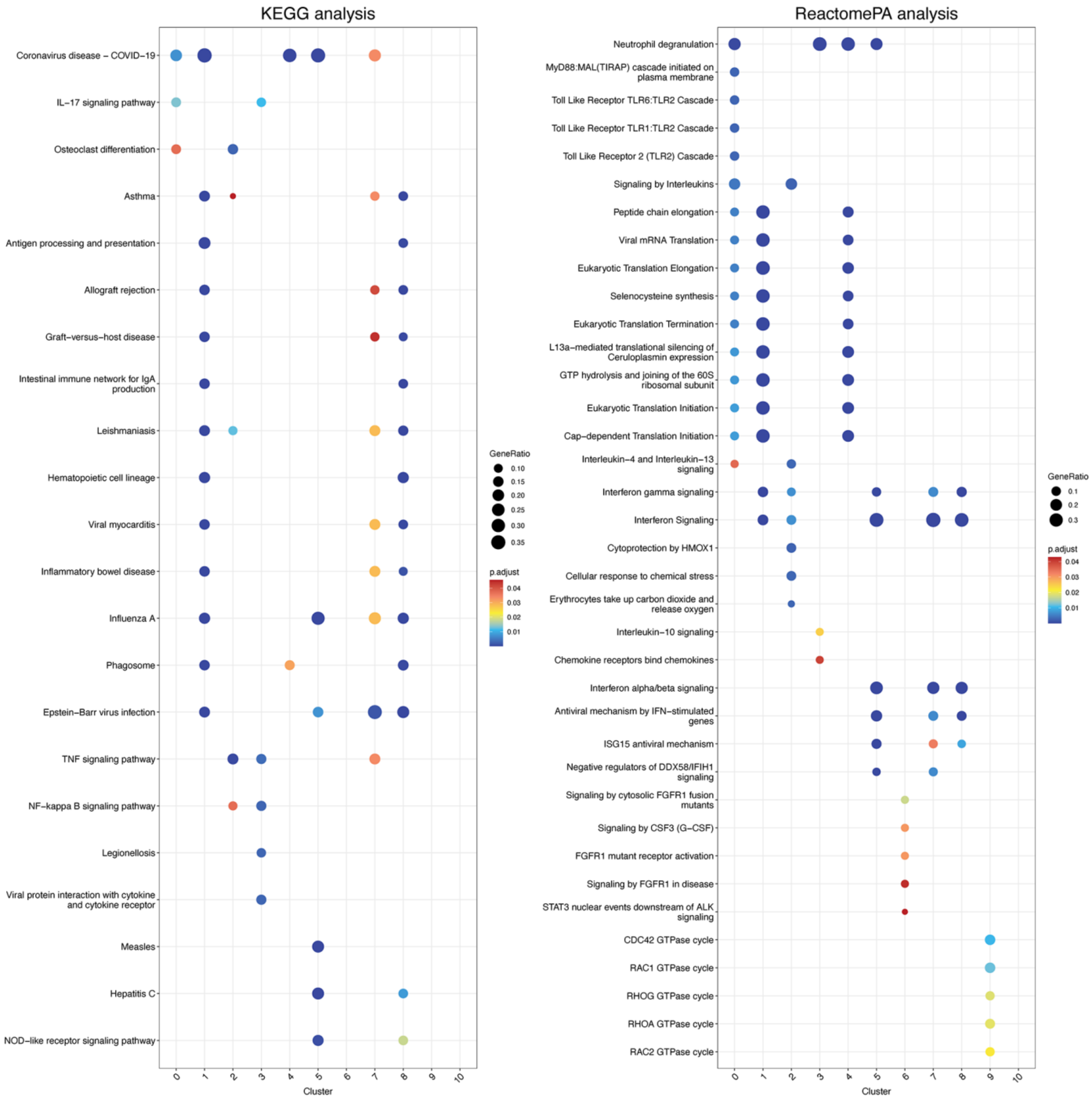

Supplementary Figure 5. KEGG enrichment analysis for all 11 subclusters of CD14^{+} monocytes. Dot plots were used.

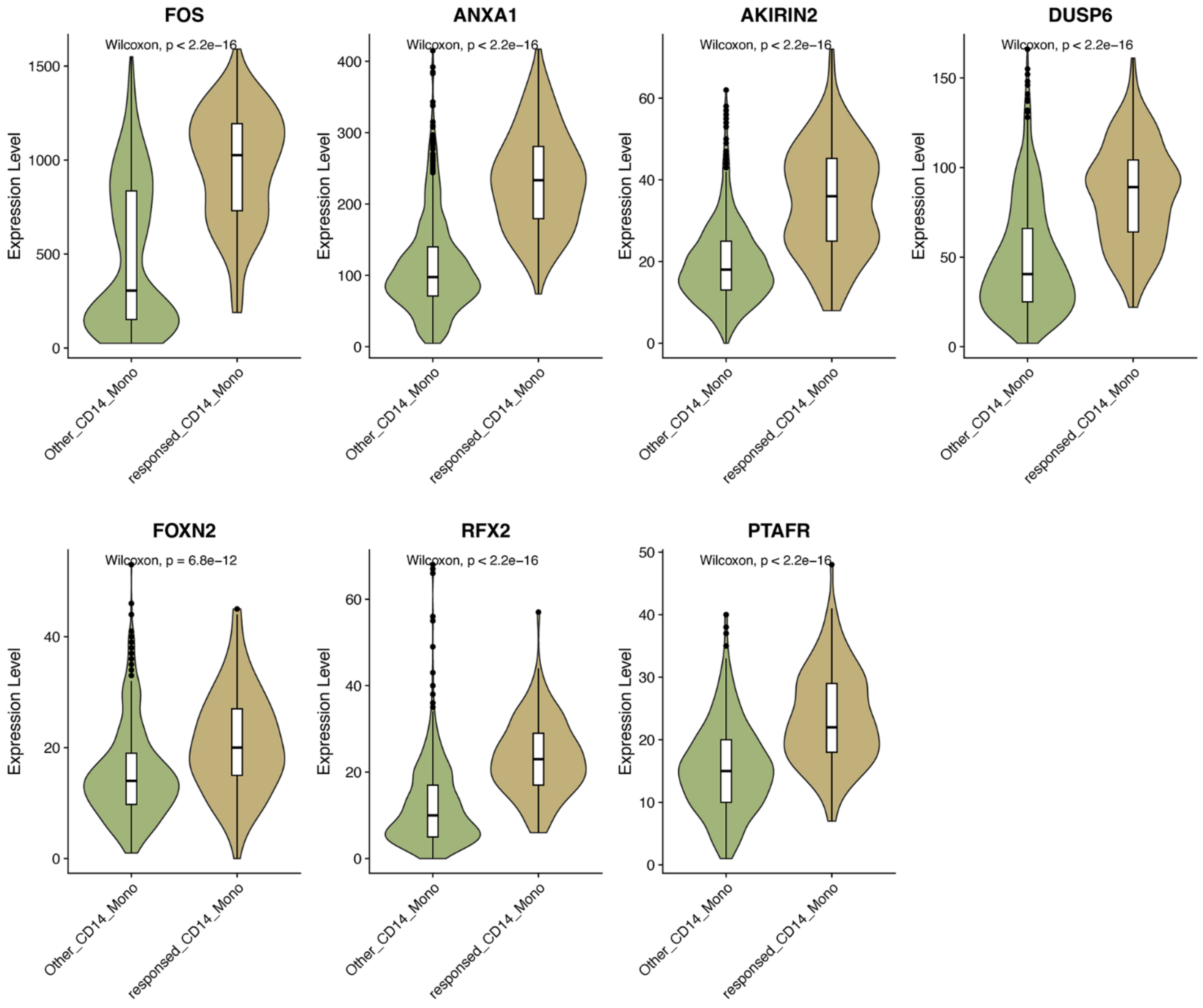

Supplementary Figure 6. Expression levels of the seven identified genes between other and response-related CD14$^+$ monocytes.

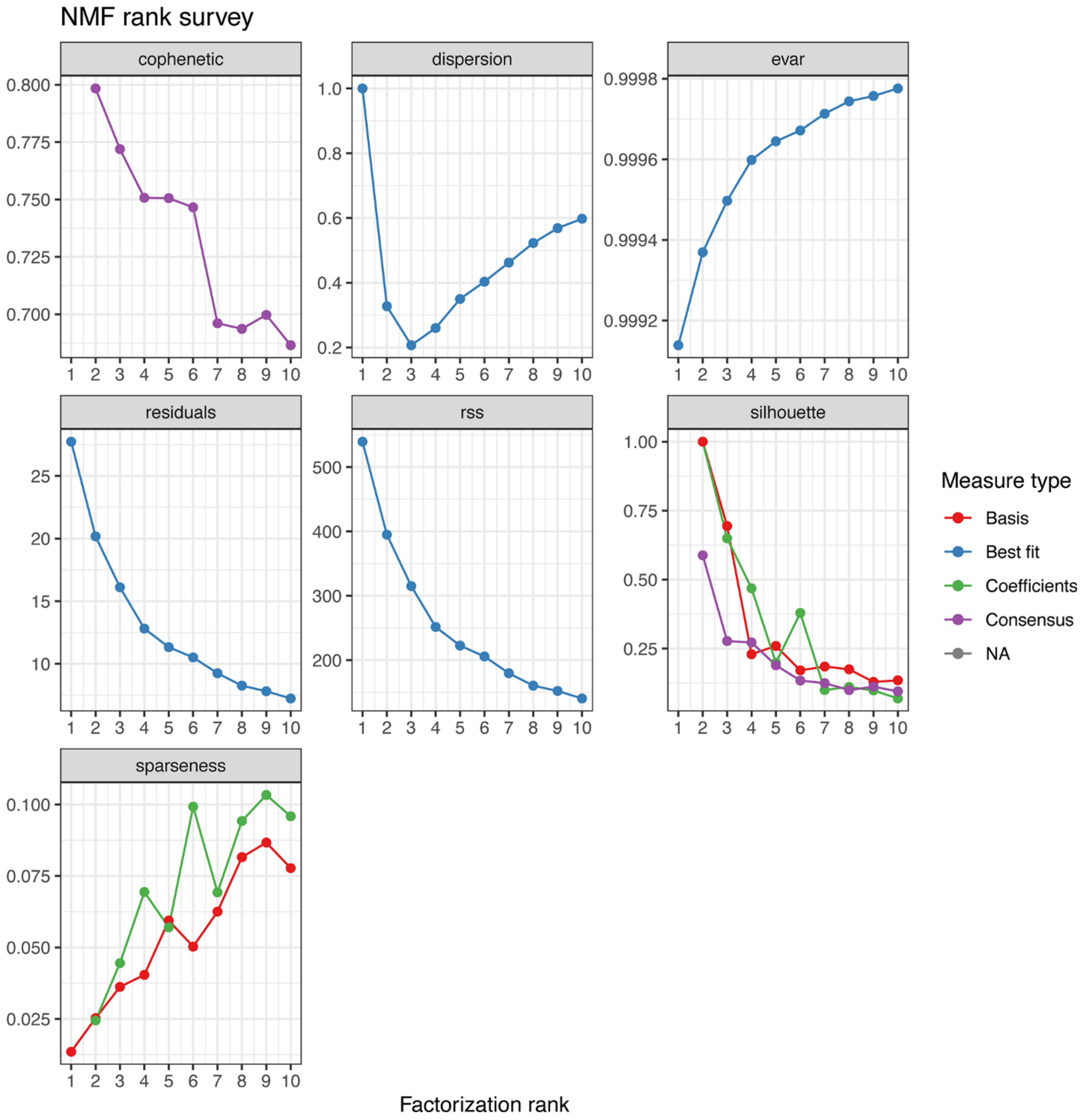

Supplementary Figure 7. The process of NMF analysis for HF patients.
