# Supplementary material for: Single cell RNA-seq identifies a FOS/JUN-related monocyte signature associated with clinical response of heart failure patients with mesenchymal stem cell therapy
Source: Aging (Albany NY). 2024 Mar 20;16(6):5651–75. doi: 10.18632/aging.205670 (PMC11006470; doi:10.18632/aging.205670)
Supplement: Supplementary Tables [file aging-16-205670-s002.pdf]

SUPPLEMENTARY TABLES

Supplementary Table 1. Analysis of changes of patients with the MSC therapy.

	Patients					
	Response (N=2)	Non-response (N=2)	Total	Test (p-value)	
	Patient 1 Patient 2	Patient 3 Patient 4			
LVEDV	3.6 (5.8)	0.1 (4.5)	1.9 (5.3)	0.2679	(Two Sample t-test)
LVESV	12.4 (2.9)	4.7 (4.9)	8.5 (5.5)	0.0078	(Two Sample t-test)
APTT	1.8 (6.5)	-7.9 (14.4)	-3.0 (11.8)	0.1611	(Two Sample t-test)
NT-proBNP	15.6 [5.0;26.8]	-21.4 [-60.5;4.1]	3.6 [-18.0;14.7]	0.0707	(Welch Two Sample t-test)
CK-MB	22.1 (13.1)	-11.0 (32.7)	5.5 (29.4)	0.0439	(Two Sample t-test)
D2	60.3 [49.4;90.4]	40.3 [36.4;51.8]	50.5 [37.4;59.6]	0.1269	(Wilcoxon rank sum test)
HSTNT	8.2 (14.4)	23.4 (17.3)	15.8 (17.1)	0.1292	(Two Sample t-test)
6WORLKJULI0M	18.2 [13.6;18.2]	2.4 [0.4;4.0]	8.4 [2.8;18.2]	0.0038	(Wilcoxon rank sum test)
LVEF	34.3 (17.9)	20.8 (7.5)	27.5 (14.9)	0.1185	(Two Sample t-test)

For normally distributed data, it is described using the mean and standard deviation.
For non-normally distributed data, it is described using the median and quartiles.
R1: Increase and decrease rates of examination and test values one month after patient cell transplantation compared to baseline.
R3: Increase and decrease rates of examination and test values three months after patient cell transplantation compared to baseline.
R6: Increase and decrease rates of examination and test values six months after patient cell transplantation compared to baseline.

Supplementary Table 2. Patient demographics.

	Overall	Response	Non-response	p-value
	N = 4	N = 2	N = 2	
Age at Diagnosis	62.25 (5.91)	60.5 (7.78)	64 (5.66)	0.658
Male	4	2	2	
Body mass index, kg/m^2	25.14 (1.2)	24.25 (1.07)	26.03 (0.06)	0.142
Alcohol use	3	2	1	
Tobacco use	1 (25%)	0 (0%)	1 (50%)	
Years since heart failure diagnosis	5.75	5	6.5	0.423
Previous myocardial infarction	1 (25%)	0 (0%)	1 (50%)	
Any type of defibrillator (AICD or CRT-D)	1 (25%)	0 (0%)	1 (50%)	
NHYA functional class	2.75 (0.5)	3 (0)	2.5 (0.71)	0.609
History of hypertension	3 (75%)	1 (50%)	2 (100%)	
History of diabetes	1 (25%)	0 (0%)	1 (50%)	
History of atrial fibrillation	1 (25%)	0 (0%)	1 (50%)	
Ventricular premature beats and ventricular tachycardia	3 (75%)	1 (50%)	2 (100%)	
HbA1c	5.98 (0.73)	5.5 (0.28)	6.45 (0.78)	0.246
Diagnosis				
CDM	3 (75%)	2 (100%)	1 (50%)	
CHD	1 (25%)	0 (0%)	1 (50%)	
Mitral regurgitation	4 (100%)	2 (100%)	2 (100%)	
Tricuspid valve regurgitation	1 (25%)	0 (0%)	1 (50%)	
Medications				
ARBs	4 (100%)	2 (100%)	2 (100%)	
Diuretic agents	1 (25%)	0 (0%)	1 (50%)	
Beta-blockers	4 (100%)	2 (100%)	2 (100%)	
Digitalis	1 (25%)	0 (0%)	1 (50%)	
Oral anticoagulants	1 (25%)	0 (0%)	1 (50%)	
Anti-platelet agents	4 (100%)	2 (100%)	2 (100%)	
SGLT-2 inhibitors	1 (25%)	0 (0%)	1 (50%)	
Statins	4 (100%)	2 (100%)	2 (100%)	
ECG				
HR	71.75 (9.25)	65 (7.07)	78.5(4.95)	0.157
QRSD (ms)	94.75(11.18)	97(15.56)	92.5(10.61)	0.768
QTJQ (ms)	395.25(37.02)	408(25.46)	382.5(53.03)	0.602
Rv5/Sv1	1.77(1.58)	3(1.18)	0.55(0.33)	0.106

For normally distributed data, it is described using the mean and standard deviation.
For non-normally distributed data, it is described using the median and quartiles.

Supplementary Table 3. Potential drugs for targeting FOS.

Chemical name	Chemical ID	CAS RN	Interaction	Interaction actions	Reference count	Organism count
Cocaine	D003042	50-36-2	Cocaine results in increased expression of FOS protein	increases^expression	31	4
Haloperidol	D006220	52-86-8	Haloperidol results in increased expression of FOS protein	increases^expression	17	3
Cadmium	D002104	7440-43-9	Cadmium results in increased expression of FOS mRNA	increases^expression	11	4
Haloperidol	D006220	52-86-8	Haloperidol results in increased expression of FOS mRNA	increases^expression	10	1
Tetrachlorodibenzodioxin	D013749	1746-01-6	Tetrachlorodibenzodioxin results in increased expression of FOS mRNA	increases^expression	10	3
Ethinyl Estradiol	D004997	57-63-6	Ethinyl Estradiol results in increased expression of FOS mRNA	increases^expression	9	5
Cyclophosphamide	D003520	50-18-0	Cyclophosphamide results in increased expression of FOS protein	increases^expression	8	1
Nitroglycerin	D005996	55-63-0	Nitroglycerin results in increased expression of FOS protein	increases^expression	8	2
Cisplatin	D002945	15663-27-1	Cisplatin results in increased expression of FOS mRNA	increases^expression	7	3
Clozapine	D003024	5786-21-0	Clozapine results in increased expression of FOS protein	increases^expression	7	3
Clozapine	D003024	5786-21-0	Clozapine results in increased expression of FOS mRNA	increases^expression	6	1
Cocaine	D003042	50-36-2	Cocaine affects the expression of FOS mRNA	affects^expression	6	2
Diethylstilbestrol	D004054	56-53-1	Diethylstilbestrol results in increased expression of FOS mRNA	increases^expression	6	2
Tetrachlorodibenzodioxin	D013749	1746-01-6	Tetrachlorodibenzodioxin results in decreased expression of FOS mRNA	decreases^expression	6	4
Cocaine	D003042	50-36-2	Cocaine affects the expression of FOS protein	affects^expression	5	2
Lipopolysaccharides	D008070		Lipopolysaccharides results in increased expression of FOS mRNA	increases^expression	5	1
Lipopolysaccharides	D008070		Lipopolysaccharides results in increased expression of FOS protein	increases^expression	5	2
Particulate Matter	D052638		Particulate Matter results in increased expression of FOS mRNA	increases^expression	5	3
Resveratrol	D000077185		Resveratrol results in increased expression of FOS mRNA	increases^expression	5	1
Cocaine	D003042	50-36-2	SCH 23390 inhibits the reaction [Cocaine results in increased expression of FOS protein]	decreases^reaction|increases^expression	4	1
Methamphetamine	D008694	537-46-2	Methamphetamine results in increased expression of FOS protein	increases^expression	4	2
Carbachol	D002217		Carbachol results in increased expression of FOS protein	increases^expression	3	1
Cisplatin	D002945	15663-27-1	Cisplatin results in increased expression of FOS protein	increases^expression	3	2
Copper	D003300	7440-50-8	Copper results in decreased expression of FOS mRNA	decreases^expression	3	2
Doxorubicin	D004317	23214-92-8	Doxorubicin results in increased expression of FOS mRNA	increases^expression	3	2
Isoproterenol	D007545	7683-59-2	Isoproterenol results in increased expression of FOS mRNA	increases^expression	3	2
Lipopolysaccharides	D008070		Lipopolysaccharides affects the localization of FOS protein	affects^localization	3	2
Lipopolysaccharides	D008070		Lipopolysaccharides results in decreased expression of FOS mRNA	decreases^expression	3	2
Lithium	D008094	7439-93-2	Lithium results in increased expression of FOS protein	increases^expression	3	1
Nitroprusside	D009599	15078-28-1	Nitroprusside affects the expression of FOS protein	affects^expression	3	1
Nitroprusside	D009599	15078-28-1	Nitroprusside results in increased expression of FOS protein	increases^expression	3	2
Ozone	D010126	10028-15-6	Ozone results in increased expression of FOS mRNA	increases^expression	3	1
Pentobarbital	D010424	76-74-4	Pentobarbital results in decreased expression of FOS protein	decreases^expression	3	1
Pentobarbital	D010424	76-74-4	Pentobarbital results in increased expression of FOS protein	increases^expression	3	1
Resveratrol	D000077185		Resveratrol results in increased expression of FOS protein	increases^expression	3	1
Tamoxifen	D013629	10540-29-1	Tamoxifen results in increased expression of FOS mRNA	increases^expression	3	3
Tretinoin	D014212	302-79-4	Tretinoin results in decreased expression of FOS mRNA	decreases^expression	3	3
